# *HMGA2* overexpression is associated with differential expression of miRNAs in uterine leiomyomas

**DOI:** 10.1186/1753-6561-7-S2-P36

**Published:** 2013-04-04

**Authors:** Julia BH Mello, Mateus C Barros-Filho, Priscila Ramos Cirilo, Fábio A Marchi, Anagloria Pontes, Silvia R Rogatto

**Affiliations:** 1 Institute of Biosciences, UNESP, Botucatu, SP, Brazil; 2 CIPE - AC Camargo Cancer Hospital, São Paulo, SP, Brazil; 3 Faculty of Medicine, UNESP, Botucatu, SP, Brazil

## Background

Uterine leiomyomas (UL) are mesenchymal benign tumors extremely common that represent a significant public health problem. The deregulation of growth factors and microRNAs (miRNAs), shortening of telomeres, excessive production of disorganized extracellular matrix, loss of heterozygosity and recurrent chromosomal aberrations (including 7q22 deletion and chromossomal rearrangements in 12q15) have been suggested to contribute to the growth of fibroids. *HMGA2*, mapped to 12q15, is a major regulator of benign tumorigenesis from mesenchyme-derived tissues and stem-cell self-renewal. In UL, *HMGA2* overexpression is frequently reported. Recently it has been shown that repression of *HMGA2* by microRNA let-7s is a critical molecular regulatory mechanism associated with tumor growth. In this study, it was evaluated three miRNAs mapped to 7q22 (miR-25, miR-93 and miR- 106b) and miR-let-7a (previously reported as *HMGA2* regulator). These findings were compared with gene expression microarray data.

## Patients and methods

Seventy-eight fresh frozen UL and 20 adjacent normal myometrium (MM) were collected from 54 patients who had undergone a hysterectomy procedure. Paired analysis were performed in 20 cases. Quantitative real time RT-PCR was applied to evaluate the expression of miR-let7a, miR-25, miR-93, and miR- 106b using RNU44, RNU48 and U47 as endogenous control. Array CGH and expression analysis was carried out using Agilent 4 x44 k arrays in the same set of cases.

## Results

Losses of 7q22 were significantly detected in UL by array CGH. In 7q22 is mapped the miR-93 and miR-106b which were found as down-regulated (p<0.001; p=0.001, respectively). miR let-7a was also down-regulated (p=0.001). Oligorrays expression analysis confirmed *HMGA2* overexpression.

## Conclusions

Losses of 7q22 were associated with miR-93 and miR-106b downexpression leading to deregulation of target genes involved in ULs pathogenesis. In addition to miR-let7a, miR-93 is also a candidate for *HMGA2* regulation in these tumors.

## Financial support

FAPESP and CNPq.

